# Assignment of the N-terminal domain of mouse cGAS

**DOI:** 10.1007/s12104-024-10213-2

**Published:** 2025-01-04

**Authors:** Hanna Aucharova, Rasmus Linser

**Affiliations:** Department of Chemistry and Chemical Biology, TU Dortmund University, Dortmund, Germany

**Keywords:** Cyclic GMP-AMP synthase, cGAS, Intrinsically disordered proteins, IDPs, NMR spectroscopy

## Abstract

Cyclic GMP-AMP synthase (cGAS) is a DNA-sensing enzyme that is a member of the nucleotidyltransferase (NTase) family and functions as a DNA sensor. The protein is comprised of a catalytic NTase core domain and an unstructured hypervariable N-terminal domain (NTD) that was reported to increase protein activity by providing an additional DNA-binding surface. We report nearly complete ^1^H, ^15^N, and ^13^C backbone chemical-shift assignments of mouse cGAS NTD (residues 5-146), obtained with a set of 3D and 4D solution NMR experiments. Analysis of the chemical-shift values confirms that the NTD is intrinsically disordered. These resonance assignments can provide the basis for further studies such as activation by DNA and protein-protein interactions.

## Biological context

cGAS is a major DNA sensor in humans and other animals that binds to double-stranded DNA and activates the STING-TBK1-IRF3 signaling axis to induce production of type I interferons and trigger innate immunity or apoptosis (Gao et al. 2013). Activation of cGAS by DNA makes it a promising drug target for autoimmune diseases and cancer (Decout et al. 2021; Gan et al. 2022). cGAS is a Mg^2+^- and Zn^2+^-binding enzyme belonging to the Mab-21-like family of nucleotidyltransferases (NTases). It was initially reported as a cytosolic DNA sensor, however, recent findings showed that the major fraction of cGAS resides in the nucleus bound to chromatin (Song et al. 2022; Jiang et al. 2019; Michalski et al. 2020), and cytoplasmic cGAS is anchored to the plasma membrane (Barnett et al. 2019). The NTD is critical for sensing nuclear chromatin, and this interaction is abrogated by multiple hyperphosphorylation at various serine and threonine residues. Chromatin-bound cGAS is kept inhibited, with its activity being maintained at a level 200-fold lower than that of cGAS stimulated with exogeneous DNA (Gentili et al. 2019). Truncation of cGAS NTD leads to cGAS mislocalization in cytoplasm and nucleus and hyper-response to genotoxic stress but a weaker response to viral infection (Barnett et al. 2019). Solution NMR studies on the cGAS N-terminal domain will enable a better understanding of its biophysical properties and associated intermolecular interactions. As a basis for such studies, we report the assignments of chemical shifts for its residues 5-146.

## Methods and experiments

### Expression and purification of cGAS NTD

The cDNA encoding *M. musculus* cGAS N-terminal domain (residues 1–146) was cloned into a pOPIN-8xHis-eGFP plasmid and overexpressed in *E. coli* RIL-X cells as an eGFP fusion protein with a PreScission protease cleavage site. To produce a uniformly ^15^N- and ^13^C-labeled protein, a single colony of *E.coli* RIL-X cells was inoculated in 1 L of a standard M9 minimal medium supplemented with 100 μg/ml ampicillin, 1 g/L ^15^NH_4_Cl, 2 g/L ^13^C-D-glucose, and 2 g/L ^15^N/^13^C-labeled ISOGRO algal extract, and cells were grown at 37 °C and 110 rpm until an OD_600_ of 0.8 was reached. The culture was chilled on ice for 20 min and expression was induced by the addition of isopropyl β-D-thiogalactopyranoside (IPTG) to a final concentration of 0.5 mM. Cells continued to grow upon shaking at 120 rpm and 20 °C for another 20 h, followed by harvesting by centrifugation at 4 °C and 4,000 g for 20 min; pellets were frozen at -80 °C until further use. Frozen cell pellets were thawed at room temperature and resuspended in lysis buffer (50 mM HEPES pH 8.0, 0.3 M NaCl, 1 mM TCEP, 1 mM PMSF, 10% (w/v) glycerol, 20 mM imidazole). After vortexing, cells were lysed with an Avestin Emulsiflex C3. Lysates were aliquoted into 45 ml tubes and centrifuged at 100,000 g and 4 °C for 20 min, then incubated at 100 °C for 10 min to precipitate cellular proteases and centrifuged again as described above. The cleared lysate was applied onto a 5 ml HisTrap HP column (GE Healthcare) using an ÄKTA Pure chromatography system (GE Healthcare) in equilibration buffer. eGFP-mNTD fusion protein was eluted with a gradient of elution buffer (50 mM HEPES pH 8.0, 0.3 M NaCl, 1 mM TCEP, 1 mM PMSF, 10% (w/v) glycerol, 0.5 M imidazole) at 150–200 mM imidazole concentration. Elution fractions were then pooled together and concentrated to 2 ml using Amicon Ultra centrifugal filters with a molecular weight cut-off (MWCO) of 30 kDa (Sigma-Aldrich). The protein was buffer-exchanged into cleavage buffer (50 mM HEPES pH 8.0, 0.3 M NaCl, 1 mM TCEP) using a PD 10 desalting column (Cytiva). The protein was then mixed with 12 mg of PreScission protease (prepared in-house) and cleavage was performed overnight at 4 °C on a rocking shaker. After cleavage, the protein mixture was mixed 1:5 (v/v) with 6 M guanidine hydrochloride (Gu-HCl) to cancel electrostatic interactions of the eGFP tag and the protein. The protein was then concentrated to 1–2 ml using Amicon Ultra centrifugal filters (MWCO 3 kDa). The protein mixture was applied onto a manually packed Ni-NTA gravity flow column (5 ml) equilibrated with equilibration buffer. A total volume of 70 ml of a flow-through fraction that contained the protein was collected. The protein was then concentrated to 1–2 ml volume and renatured by rapid dilution with renaturing buffer at a ratio of 1:10 (v/v). The protein was then concentrated again to a final volume of 2 ml and exchanged into NMR buffer (20 mM MES pH 6.0, 0.1 M NaCl, 5 mM TCEP, 1 mM PMSF). For preparation of NMR samples, 270 μl of protein solution was mixed with D_2_O to a final concentration of 10% (v/v) and 0.1 M DSS to a final concentration of 1–3 mM.

### NMR spectroscopy

An approximately 1 mM uniformly ^15^N/^13^C-labeled mNTD sample in NMR buffer was used for resonance assignments. DSS was used for direct ^1^H chemical shift referencing, whereas ^13^C and ^15^N chemical shifts were indirectly referenced. Protein backbone resonances were assigned via standard 2D HSQC, 3D HNCO, 3D HNCA, 3D HN(CO)CA, 3D HN(CA)CO, and 3D CBCANH experiments, as well as a 4D HN(COCA)NH (Bracken et al. 1997) experiment. All NMR experiments were recorded at 25 °C on a Bruker Avance III HD 800 MHz spectrometer equipped with a ^2^H, ^1^H, ^13^C, ^15^N four-channel cryogenic TCI probe. The 4D HN(COCA)NH experiment was recorded using non-uniform sampling with 0.1% sampling density and reconstructed using the SSA algorithm (Stanek and Koźmiński 2010). NMR data were processed using TopSpin (Bruker) and nmrPipe (NIST IBBR) and analyzed using CCPNMR Analysis v. 3.2.2 (Skinner et al. 2016). Automated sequence assignment was performed in FLYA (Schmidt and Güntert 2012), and prediction of chemical shifts was done in POTENCI (Nielsen and Mulder 2018).

### Extent of assignments and data deposition

**Fig. 1:**
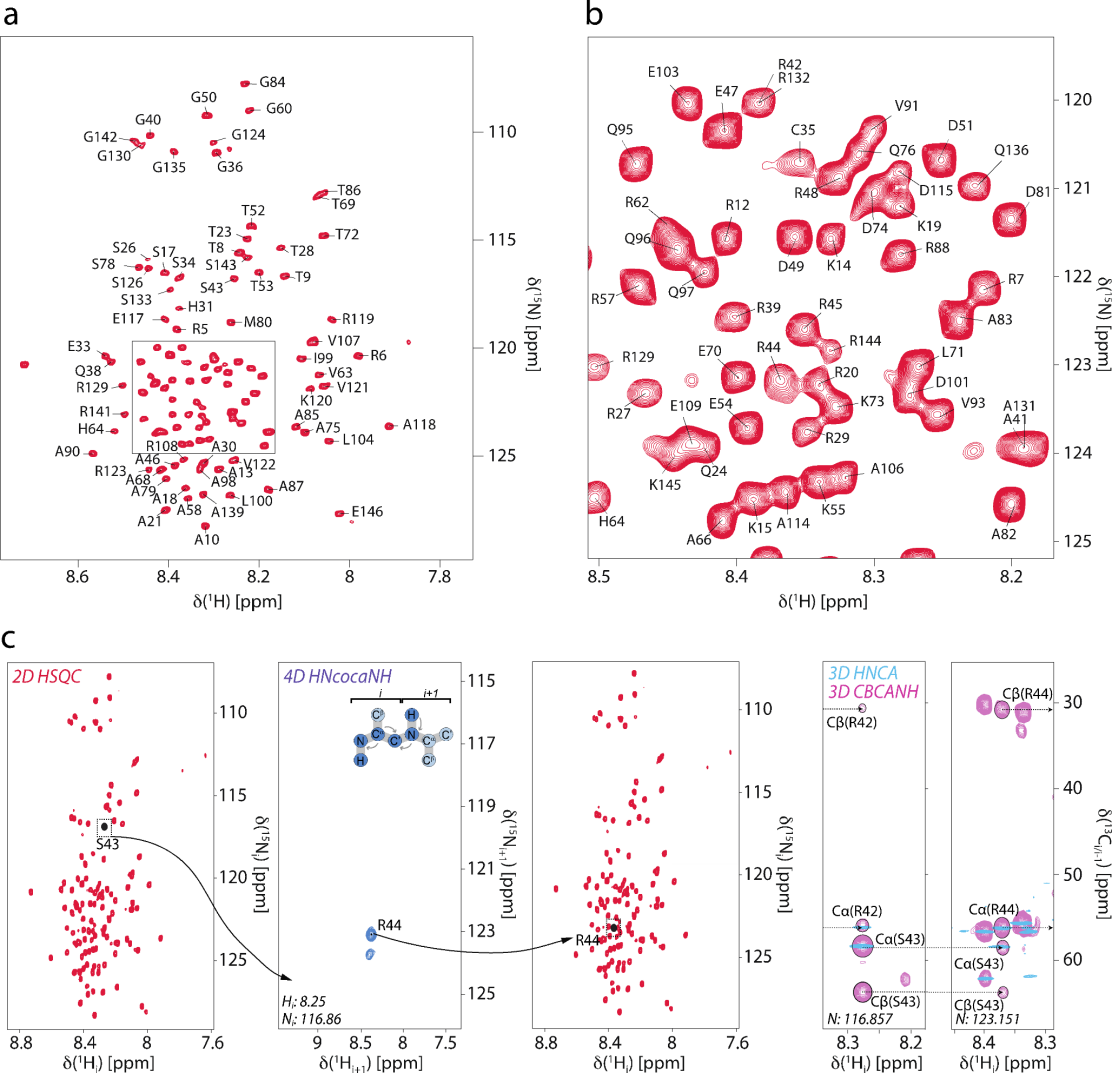
(**a**) Assigned 2D ^1^H-^15^N HSQC of mouse cGAS N-terminal domain, recorded at 800 MHz ^1^H Larmor frequency at 25°C. (**b**) Excerpt from the most crowded region of the HSQC as marked by the black box in (a). (**c**) Representation of the assignment process using the 4D HN(COCA)NH experiment. The process starts with choosing a reasonably well-separated peak in the 2D HSQC and navigating to the 3D HNCA/CBCANH window to identify the amino acids types of residues *i* and *i + 1* based on C^*β*^ and C^*α*^ chemical shifts. Navigating from the 2D HSQC to the 4D HN(COCA)NH window will usually result in seeing exactly one peak corresponding to amino acid *i + 1*. The connectivity can then be confirmed by comparison of C^*α*^, C^*β*^, and C’ chemical shifts between complementary 3D experiments

The mNTD ^1^H-^15^N HSQC spectrum displays limited peak dispersion, with ^1^H^N^ chemical shifts clustered between 7.7 and 8.7 ppm, indicating a predominantly disordered structure (Fig. 1). An initial set of ^1^H-detected 3D experiments suffered from signal overlap in the carbon dimension, and the resonances could hardly be assigned unambiguously without the 4D HN(COCA)NH spectrum. This experiment benefits from the long *T*_*2*_ time of IDPs and allows to obtain unambiguous sequential assignments in a reasonably short time. Automated sequence assignment in FLYA resulted in 77% assignments of non-proline residues, which was then improved to 92% by manual assignment in combination with chemical shift prediction from POTENCI (Fig. 1a/b). Figure 1c represents the sequential connectivity through the 4D HN(COCA)NH experiment as well as the carbon chemical shifts for residues *i* and *i + 1* for an exemplary amide peak.

**Fig. 2:**
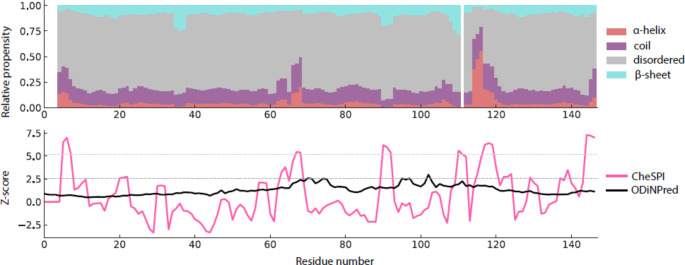
Secondary structural propensities of mouse cGAS NTD according to CheSPI, in comparison with secondary-structure Z-score calculation of the primary sequence obtained via ODiNPred (Dass et al. 2020)

The backbone N/H resonances are completely assigned other than residues Met1, Glu2, Asp3, Gln111, His127, Arg128, and Arg138. The protein primary sequence possesses two repeats, bearing the amino acid motif RGARS, in positions 39–43 and 129–133. These repeats show overlapping signals for residues Ala41 and Ala131, as well as for residues Arg42 and Arg132, which complicates unambiguous assignment. The number of assignments made for individual types of nuclei is: ^1^H^N^: 113, ^15^N^H^: 113, ^13^C^*α*^: 131, ^13^C^*β*^: 120, and ^13^C’: 134. The protein sequence contains 146 amino acids, of which ten are Gly (having no ^13^C^*β*^) and 22 are Pro (lacking ^1^H^N^ and yielding no ^15^N^H^ assignments in the triple-resonance experiments). Consequently, 92% of back- bone ^15^N and ^1^H resonances could be assigned and, excluding proline residues, 89% of ^13^C^*α*^, 96% of ^13^C^*β*^, and 91% of ^13^C’ resonances could be assigned. By using C^*α*^ and C^*β*^ NMR chemical shifts (∆δ(C^*α*^) − ∆δ(C^*β*^), we determined the deviation from random-coil chemical shift values to identify potential α-helices, β-sheets, and disordered loops. Analysis via CheSPI (Nielsen and Mulder 2021) showed similar overall structural propensities, which indicate the probability of the protein to be mostly in random-coil conformation with short transient α-helices around residues 115 and 120 (Fig. 2, top). To address proline cis/trans isomerization, we have recorded a dedicated, carbon-detected experiment (^13^C-CON), in which we do not see extra peaks in the proline region. In the triple-resonance experiments, no extra peaks hinting to alternative proline confirmations were confidently detected either. On the basis of the signal-to-noise ratio obtained, we can hence exclude major secondary (cis) proline populations (> around 10% or higher). The assiged proline shifts (major conformer) all have a ^13^C^*β*^ chemical shift of around 32 ppm and agree very well with (trans) chemical shifts predicted by ncIDP (Tamiola et al. 2010). Minor cis proline populations, however, are still likely.

The profile of Z-scores derived from the experimental NMR data suggests the presence of five regions of decreased mobility within the NTD: Arg5-Arg8, Ala66-Ala68, Ala90-Arg92, Pro110-Arg119, and Lys145-Pro147 (Fig. 2, bottom). Although the NTD is largely disordered, our analysis suggests some residual order spanning the entire protein. The lower part of Fig. 2 also includes assessment of the Z-score purely on the basis of sequence, taking the ODINpred framework (Dass et al. 2020) into account.

## Data Availability

The reported ^1^H, ^13^C and ^15^N chemical-shift assignments of the cGAS N-terminus from mus musculus (residues 5 - 146) at pH 6.0 and 25 °C have been deposited in the Biological Magnetic Resonance Data Bank (BMRB, http://www.bmrb.wisc.edu/) under the BMRB accession number 52649.

## References

[CR1] Barnett KC, Coronas-Serna JM, Zhou W, Ernandes MJ, Cao A, Kranzusch PJ, Kagan JC (2019) Phosphoinositide interactions position cGAS at the plasma membrane to ensure efficient distinction between self- and viral DNA. Cell 176:1432–1446e11. 10.1016/j.cell.2019.01.049 30827685 10.1016/j.cell.2019.01.049 PMC6697112

[CR2] Bracken C, Palmer AG, Cavanagh J (1997) (H)N(COCA)NH and HN(COCA)NH experiments for 1H-15 N backbone assignments in 13 C/15 N-labeled proteins. J Biomol NMR 9:94–100. 10.1023/A:1018679819693/METRICS 9081546 10.1023/a:1018679819693

[CR3] Dass R, Mulder FAA, Nielsen JT (2020) ODiNPred: comprehensive prediction of protein order and disorder. Sci Rep 2020 10:1(10):1–16. 10.1038/s41598-020-71716-1 10.1038/s41598-020-71716-1 PMC7479119 32901090

[CR4] Decout A, Katz JD, Venkatraman S, Ablasser A (2021) The cGAS-STING pathway as a therapeutic target in inflammatory diseases. Nat Rev Immunol 21:548–569. 10.1038/S41577-021-00524-Z 33833439 10.1038/s41577-021-00524-z PMC8029610

[CR5] Gan Y, Li X, Han S, Liang Q, Ma X, Rong P, Wang W, Li W (2022) The cGAS/STING pathway: a novel target for cancer therapy. Front Immunol 12:795401. 10.3389/FIMMU.2021.795401 35046953 10.3389/fimmu.2021.795401 PMC8761794

[CR6] Gao P, Ascano M, Wu Y, Barchet W, Gaffney BL, Zillinger T, Serganov AA, Liu Y, Jones RA, Hartmann G, Tuschl T, Patel DJ (2013) Cyclic [G(2’,5’)pA(3’,5’)p] is the metazoan second messenger produced by DNA-activated cyclic GMP-AMP synthase. Cell 153:1094–1107. 10.1016/J.CELL.2013.04.046 23647843 10.1016/j.cell.2013.04.046 PMC4382009

[CR7] Gentili M, Lahaye X, Nadalin F, Nader GFP, Puig Lombardi E, Herve S, De Silva NS, Rookhuizen DC, Zueva E, Goudot C, Maurin M, Bochnakian A, Amigorena S, Piel M, Fachinetti D, Londoño-Vallejo A, Manel N (2019) The N-terminal domain of cGAS determines preferential association with centromeric DNA and innate immune activation in the nucleus. Cell Rep 26:2377–2393e13. 10.1016/j.celrep.2019.01.105 30811988 10.1016/j.celrep.2019.01.105 PMC6391843

[CR8] Jiang H, Xue X, Panda S, Kawale A, Hooy RM, Liang F, Sohn J, Sung P, Gekara NO (2019) Chromatin-bound cGAS is an inhibitor of DNA repair and hence accelerates genome destabilization and cell death. EMBO J 38(21). 10.15252/EMBJ.2019102718 10.15252/embj.2019102718 PMC6826206 31544964

[CR9] Michalski S, de Oliveira Mann CC, Stafford C, Witte G, Bartho J, Lammens K, Hornung V, Hopfner KP (2020) Structural basis for sequestration and autoinhibition of cGAS by chromatin. Nature. 10.1038/s41586-020-2748-0 10.1038/s41586-020-2748-0 32911480

[CR10] Nielsen JT, Mulder FAA (2018) Potenci: prediction of temperature, neighbor and ph-corrected chemical shifts for intrinsically disordered proteins. J Biomol NMR 70:141–165. 10.1007/S10858-018-0166-5 29399725 10.1007/s10858-018-0166-5

[CR11] Nielsen JT, Mulder FAA (2021) CheSPI: chemical shift secondary structure population inference. J Biomol NMR 75:273–291. 10.1007/S10858-021-00374-W 34146207 10.1007/s10858-021-00374-w

[CR12] Schmidt E, Güntert P (2012) A new algorithm for reliable and general NMR resonance assignment. J Am Chem Soc 134:12817–12829. 10.1021/JA305091N 22794163 10.1021/ja305091n

[CR13] Skinner SP, Fogh RH, Boucher W, Ragan TJ, Mureddu LG, Vuister GW (2016) CcpNmr AnalysisAssign: a flexible platform for integrated NMR analysis. J Biomol NMR 66:111–124. 10.1007/S10858-016-0060-Y 27663422 10.1007/s10858-016-0060-y PMC5095159

[CR14] Song JX, Villagomes D, Zhao H, Zhu M (2022) cGAS in nucleus: the link between immune response and DNA damage repair. Front Immunol 13:1076784. 10.3389/fimmu.2022.1076784 10.3389/fimmu.2022.1076784 PMC9797516 36591232

[CR15] Stanek J, Koźmiński W (2010) Iterative algorithm of discrete fourier transform for processing randomly sampled NMR data sets. J Biomol NMR 47:65–77. 10.1007/S10858-010-9411-2 20372976 10.1007/s10858-010-9411-2

[CR16] Tamiola K, Acar B, Mulder FA (2010) Sequence-specific random coil chemical shifts of intrinsically disordered proteins. J Am Chem Soc 132:18000–18003. 10.1021/ja105656t 21128621 10.1021/ja105656t

